# Journal impact factor, trial effect size, and methodological quality appear scantly related: a systematic review and meta-analysis

**DOI:** 10.1186/s13643-020-01305-w

**Published:** 2020-03-09

**Authors:** Michael Saginur, Dean Fergusson, Tinghua Zhang, Karen Yeates, Tim Ramsay, George Wells, David Moher

**Affiliations:** 1 Montfort Research Institute, 713 Montreal Road, Ottawa, Canada; 2 Ottawa Hospital Research Institute, 501 Smyth Road, Ottawa, K1H 8L6 Canada; 3 Department of Medicine, Queen’s University, 76 Stuart Street, Kingston, K7L 2V7 Canada; 4 University of Ottawa Heart Institute, 40 Ruskin St, Ottawa, ON K1Y 4W7 Canada

**Keywords:** Publication bias, Journal impact factor, Systematic review, Meta-analysis, Clinical trial, Research design

## Abstract

**Background:**

As systematic reviews’ limited coverage of the medical literature necessitates decision-making based on unsystematic review, we investigated a possible advantage of systematic review (aside from dataset size and systematic analysis): does systematic review avoid potential bias in sampling primary studies from high impact factor journals? If randomized controlled trials (RCTs) reported in higher-impact journals present different treatment benefits than RCTs reported in lower-impact journals, readers who focus on higher-impact journals for their rapid literature reviews may introduce bias which could be mitigated by complete, systematic sampling.

**Methods:**

We randomly sampled Cochrane Library (20 July 2005) treatment reviews that measured mortality as a binary outcome, published in English or French, with at least five RCTs with one or more deaths. Our domain-based assessment of risk of bias included funding source, randomness of allocation sequence, blinding, and allocation concealment. The primary analysis employed logistic regression by a generalized linear model with a generalized estimating equation to estimate the association between various factors and publication in a journal with a high journal impact factor (JIF).

**Results:**

From the 29 included systematic reviews, 189 RCTs contributed data. However, in the primary analyses comparing RCT results within meta-analyses, there was no statistically significant association: unadjusted odds of greater than 50% mortality protection in high-JIF (> 5) journals were 1.4 (95% CI 0.42, 4.4) and adjusted, 2.5 (95% CI 0.6, 10). Elements of study quality were weakly, inconsistently, and not statistically significantly correlated with journal impact factor.

**Conclusions:**

Journal impact factor may have little to no association with study results, or methodological quality, but the evidence is very uncertain.

## Background

Most [1–5] but not all experts [6–8] recommend systematic review as the most authoritative information source. On a per-study basis, systematic reviews are cited more often than primary studies [9], but they cover a limited number of topics [10]. The frequent, often necessary use of incomplete review despite epidemiologists’ preference for systematic reviews begs a question about the “value added” by a systematic review. Recognized advantages of systematic reviews include limiting opaque and inappropriate retrospective data review, obtaining a larger sample [11], and exploring publication bias qualitatively. Does systematic review also avoid bias in selecting a sample to read up on the field of medicine [12, 13]? If randomized controlled trials (RCTs) reported in higher-impact journals present different treatment benefits than RCTs reported in lower-impact journals, then unsystematic inclusion of higher- over lower-impact journals from their rapid literature reviews may introduce bias, whereas the systematic review’s complete sample frame would protect against biased reading. Conversely, if there is no significant relationship between journal impact factor (JIF) and effect size estimates, there would be no evidence to support systematic sampling of all studies to avoid bias in the selection of RCTs: a lack of association would support a greater trust in the primary data underpinning lay and rapid literature reviews (albeit with the caveats mentioned above in terms of data interpretation). Thus, the primary purpose of this study was to determine whether clinical trials’ effect sizes are associated with JIF.

If higher-JIF trials were also of higher-quality and at lower risk of bias by design, then their results would be more valid, independent of the quantitative association between JIF and effect size. This makes study quality not only a potential confounder in the relationship between JIF and trial validity [14, 15], but also relevant in the reader’s selection of primary studies to review. Therefore, we investigated as a secondary objective whether elements of study quality were associated with publication in higher impact journals.

## Materials and methods

### Identification and selection of relevant studies

We used a multi-stage sampling strategy to identify systematic reviews that reported on mortality as an outcome. We limited this review to the mortality outcome due to its simplicity, universality, and reliability, in order to limit confounding related to inter-study differences in measurement. Using an electronic literature search of the Cochrane Library (20 July 2005), we created a numbered list of potentially relevant systematic reviews: reviews with mortality, survival, death, casualty, or longevity in the title, abstract, or keywords. For the pilot, we randomly selected systematic reviews until we obtained two eligible systematic reviews that met our inclusion criteria. Then, a separate, computer-generated list identified the remainder of the sample. Selection criteria are described in Table 1. When a systematic review included multiple eligible meta-analyses, two authors (KY, MS) selected one meta-analysis based on the pre-articulated principles of what they considered most clinically relevant. RCTs were compared within meta-analyses, as there they were independently matched by the systematic reviewers for clinical and methodological homogeneity (which reduces confounding). When there were multiple study publications for a given RCT, the “primary” journal publication was the first complete report which reported on at least 85% of the total patients and a primary study outcome.

**Table 1:** Selection criteria

a) Selection criteria for inclusion of systematic reviews	
Human	
English or French	
Binary mortality outcome	
5 to 30 eligible RCTs with at least one death	
b) Selection criteria for RCT inclusion	
Design	Patient-level RCT (excluded if quasi-randomized or cluster randomized*)
Population	Human
Intervention	Any
Control	Any
Outcome	Mortality
	Numerator/denominator in each group
Journal	Journal impact factor ascertainable

*Quasi-randomized, e.g., alternating allocation, allocation based on chart number, etc.; re: cluster-randomization, an exception was made for studies which randomized mothers and counted neonatal deaths

### Description of systematic reviews and RCTs

MS extracted the following data about the systematic reviews: date of most recent substantive amendment, clinical area, type of control, and number of systematic review extractors. At the RCT level, MS used every obtainable cited trial report to check the RCT-related data published in Cochrane. RCT characteristics not presented in the Cochrane reviews were extracted directly from the primary publications.

Cochrane always published data on mortality, on which journal(s) published the RCT, and on grades of allocation concealment. Other RCT data included in this review were journal of publication of the primary report^1^, country of study origin^2^, number of recruiting centers, funding source, randomness of the allocation sequence, blinding, number randomized, number analyzed, and analytical use of intent-to-treat. We used Web of Science JIF from 1993 (closest to the median year of publication and modeled as a continuous variable and dichotomized [14, 15] into > 5 or ≤ 5, substituted by 2008 JIF in the 6% of RCTs where 1993 JIF was unavailable, not the 5-year JIF which had more missing data).

Assignment of grades of allocation concealment was determined by the 2006 Cochrane handbook [16]—for the purpose of this study, equivalent to the latest version [17], with one extension. Our change was our assignment of a “D” grade of allocation concealment (“not done”) when no method of allocation concealment was described, as opposed to a “B” (unclear). This distinguished RCTs with no description of allocation concealment from RCTs that described a partial method of allocation concealment (e.g., a “B” from sealed envelopes) and reflected the observation that not reporting allocation concealment usually reflects the lack of a defined protocol for allocation concealment [15].

Disagreements between MS and the authors of the systematic reviews were resolved with a second author’s opinion: DF or DM on methodology and KY on medicine. JIFs were applied only after the other data was extracted, initially on a separate spreadsheet. Calculations were deferred until after data collection was complete.

### Statistical analysis

First, the unadjusted associations between JIF and RCT statistical significance were considered across all studies, not clustered with other RCTs from the same meta-analyses. Single-predictor logistic regression models used Stata 12.1, to model JIF as a predictor of statistically significant RCT mortality differences: tests for the statistical significance (*p* < 0.05) of each RCT employed a *Z*-test calculator for comparison of two proportions [18].

The primary analysis described the relationship of effect size and other predictor variables with the outcome of a high JIF (> 5), with odds ratios, *p* values, and 95% confidence intervals (unadjusted in Table 4 and adjusted in Table 5). A logistic regression by a generalized linear model with a generalized estimating equation was used to estimate the parameters considering a possible unknown correlation within a systematic review. An odds ratio greater than 1 suggested increased odds of higher JIF. SAS version 9.3 was used to generate descriptive statistics and for the primary analysis (by SAS Institute Inc. Cary, NC, USA.).

Secondary analyses employed multiple linear regression to determine whether or not the JIF was predictive of the effect size: the effect size measured as relative risk of mortality, standardized so that all relative risks were less than or equal to 1 (Table 6).

## Results

**Fig. 1:**
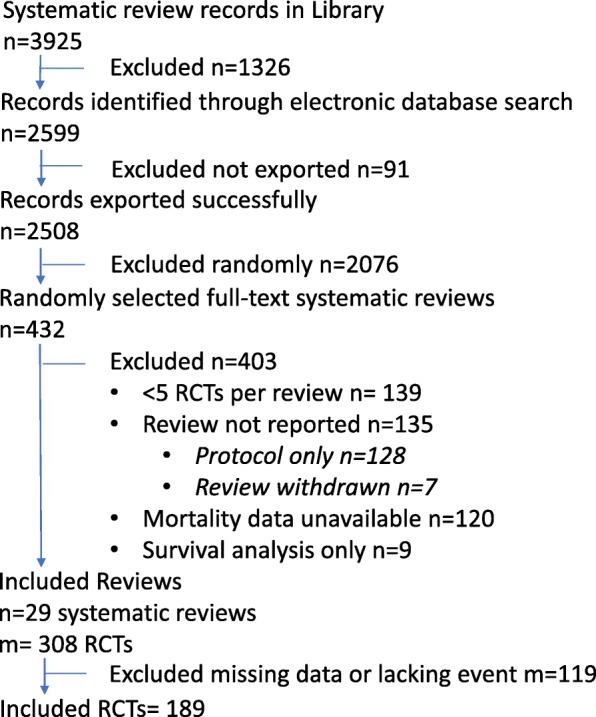
PRISMA flow diagram

From a random sample of 430 of the potentially relevant systematic reviews, 29 met our full eligibility criteria. The most common reasons for systematic review exclusion were having fewer than 5 RCTs with a death [19] per review (32%), the review lacking data on mortality (28%), and the entire review not being reported (31% of the total, of which 95% were published review protocols and 5% were reviews that had been withdrawn). See Fig. 1 for the PRISMA flow diagram (and for the PRISMA checklist, Additional file 1).

The characteristics of the included systematic reviews and trials are listed in Tables 2 and 3. Most reviews (93%) employed dual data extraction. Thirty-seven percent of reviews compared two active treatments and covered a variety of clinical topic areas, but primarily adult medicine.

**Table 2:** Characteristics of systematic reviews (*n* = 29)

Systematic reviews	
RCTs included per review, mean	7
Publication date, mean (range)	2004 (1998 to 2008)
2 or more independent data extractors	93%
Active control	37%
Topic areas	
Medicine	59%
Surgery (including anesthesia)	15%
Pediatrics	15%
Obstetrics	11%

**Table 3:** Characteristics of trials (*n* = 189)

Mortality primary outcome	10%
Statistically significant for mortality	9%
Mortality reported in primary paper	98%
Multicenter	37%
Centers, mean number	10
Domain	
Medicine	43%
Obstetrics	14%
Pediatrics	21%
Surgery	22%
Country	
Canada/USA	33%
Europe	53%
Asia	4%
Other	11%
Random sequence	
Unclear	54%
Y	46%
N	1%
Allocation concealment, grade	
Adequate (A)	34%
Unclear (B)	27%
Inadequate (C)	2%
Not Used (D)	37%
Double-blind	39%
Analysis	
Unclear	30%
As treated	29%
As randomized	41%
Publication year, mean	1993
External funding	
Peer review	47%
Industry	42%
Not stated	11%
Patients in analysis, mean number (median)	563 (165)
Deaths, mean number (median)	56 (21)

Of the 308 potentially eligible RCTs, 189 were included after exclusions for missing data (e.g., JIF unavailable for a French journal) or for lacking an event in one of the trial arms. Of the 189 included trials, only 10% defined mortality as the primary outcome, but 98% reported mortality data in the primary paper. With regard to RCT internal validity, 47% included a description of truly random sequence generation, 36% double-blinding, and 30% adequate allocation concealment. Seventy percent of studies reported funding either from a peer-review (47%) and/or industry (30%) source. The mean RCT publication year was 1993, 11 years prior to the average publication year of the systematic reviews.

First, the associations between JIF and statistical significance were considered across all studies: not matched with other RCTs from the same meta-analysis and not adjusted for study quality. Then, JIF was a statistically significant positive predictor of a statistically significant difference in mortality rates: with an odds ratio of 1.09 per unit of JIF (*p* = 0.002) or 4.4 per log-transformed JIF unit (*p* = 0.004). However, the primary analyses compared RCT results within meta-analyses and adjusted for confounders such as study quality: in these models, there was no statistically significant association. In the primary model, the odds of greater than 50% mortality protection in high JIF journals were 1.4 (95% CI 0.42, 4.4.; Table 4) and in the adjusted model 2.5 (95% CI 0.6, 10; Table 5). In the secondary analysis, the relative decrease in mortality rates increased 1.4% for each unit of JIF (95% CI of the relative odds 0.96, 1.02; Table 6).

**Table 4:** Univariate predictors of high impact factor (> 5)

Potential predictors	Odds ratio (95% CI)	*p* value
Effect size (relative risk)		0.66
0–0.5	1.367 (0.423, 4.415)	
0.5–1	Ref	
> 1	0.774 (0.399, 1.501)	
Statistically significant	2.667 (0.962, 7.390)	0.06
Mortality is primary outcome	1.671 (0.554, 5.038)	0.36
Multicenter	2.880 (1.183, 7.011)	0.02
Medical domain		0.07
Medicine	6.872 (1.180, 40.027)	
Obstetrics	3.325 (0.264, 41.731)	
Pediatrics	1.727 (0.3096, 9.636)	
Surgery	Ref	
Any industry funding	2.595 (1.102, 6.111)	0.03
Peer review funding	1.275 (0.489, 3.324)	0.62
Random sequence	1.352 (0.641, 2.852)	0.43
Allocation concealment, clearly adequate (A) or not (BCD)	1.188 (0.616, 2.288)	0.61
Double-blind	1.564 (0.544, 4.503)	0.41
Analysis as randomized		0.09
Unclear	0.427 (0.158, 1.155)	
As treated	0.320 (0.131, 0.783)	
As randomized	Ref	
Sample size (every 10 increase)	1.014 (1.002, 1.026)	0.02
Country		0.84
Canada/USA	2.651 (0.257, 27.376)	
Europe	2.108 (0.213, 20.864)	
Asia	Ref	
Other	2.000 (0.183, 21.861)	

*CI* confidence interval

**Table 5:** Multi-predictor model of high impact factor (> 5)

Potential predictors	Odds ratio (95% CI)	*p* value
Effect size (relative risk)		0.43
0–0.5	2.48 (0.61, 10.1)	
0.5–1	Ref	
> 1	0.79 (0.32, 1.91)	
Statistically significant	1.50 (0.47, 4.84)	0.50
Multicenter	1.34 (0.68, 2.62)	0.39
Medical domain		0.11
Medicine	4.25 (0.82, 22.2)	
Obstetric	0.68 (0.06, 8.03)	
Pediatrics	0.99 (0.17, 5.71)	
Surgery	Ref	
Industry funding	1.61 (0.71, 3.67)	0.26
Peer review funding	0.99 (0.38, 2.57)	0.99
Random sequence	1.58 (0.63, 3.93)	0.33
Allocation concealment, clearly adequate (A) or not (BCD)	0.53 (0.26, 1.08)	0.08
Double-blind	0.88 (0.21, 3.64)	0.86
Analysis as randomized		0.65
Unclear	0.60 (0.17, 2.09)	
As treated	0.57 (0.18, 1.80)	
As randomized	Ref	
Sample size (every 10 increase)	1.012 (0.997, 1.027)	0.12
Country		0.84
Canada/USA	2.60 (0.11, 63.0)	
Europe	1.54 (0.06, 36.6)	
Asia	Ref	
Other	2.42 (0.07, 84.7)	

*CI* confidence interval

**Table 6:** Multi-predictor model of mortality risk

Predictor	Odds ratio (95% CI)*	*p* value
Impact factor in 1993 (per unit of impact factor)	0.986 (0.96, 1.02)	0.37
Mortality is primary outcome	0.62 (0.31, 1.25)	0.18
Mortality in primary paper	1.25 (0.67, 2.33)	0.48
Centers, number	0.997 (0.98, 1.01)	0.64
Industry funding	0.93 (0.60, 1.43)	0.73
Peer review funding	1.08 (0.74, 1.59)	0.69
Random sequence	0.87 (0.59, 1.28)	0.47
Allocation concealed, clearly adequate (A) or not (BCD)	1.42 (0.91, 2.20)	0.12
Double-blind	0.76 (0.48, 1.21)	0.25
Analysis as randomized	0.96 (0.56, 1.64)	0.88
Sample size	1.000076 (0.99989, 1.00026)	0.43
Europe	0.95 (0.60, 1.49)	0.81
Asia	1.02 (0.43, 2.39)	0.97
Other non-USA/Canada	0.80 (0.38, 1.71)	0.57

*CI* confidence interval

In the primary model, statistically significant individual predictors of high JIF were larger sample sizes (OR 1.014 per ten subjects), multiple study centers (OR 2.9), and industry funding (OR 2.6). Also, in the primary model, *p* values between 0.05 and 0.07 were observed for the individual predictors “statistically significant mortality outcome (OR 2.7)” and “medical domain.” These associations were not statistically significant in the primary multi-predictor model.

The trends toward increased rates of publication of trials with safeguards against bias such as allocation concealment, truly random sequence generation, and double-blinding in higher-impact reports had *p* values of association between 0.4 and 0.65 (Table 4); with adjustment for other predictors, however, the adjusted odds of clearly adequate allocation concealment in a high JIF journal were lower, 0.53 (95% CI 0.26, 1.08; Table 5). Mortality being a primary outcome was associated with a trend toward higher odds of publication in a higher-impact journal (OR 1.7, 95% CI 0.55, 5.0), but a smaller effect size (relative odds 0.62, *p* = 0.18).

## Discussion

This systematic review is novel in having investigated the association between JIF and study results while adjusting for potential confounders and items that may introduce bias. Confounding limits the prior relevant investigations. In one study, a secondary analysis found no statistically significant association of JIF and RCT conclusions [20]; however, reviews of research proposals [21] and of conference submissions [20] found tendencies toward lower journal impact factor among statistically negative studies [21, 22]. Unfortunately, these reviews did not match studies by clinical questions while separating effect size estimates from testing for statistical significance [22–24]. A review of highly cited clinical research studies did find that the evidence they presented for trial interventions was more positive than studies published later on the same topic [23]; however, this conflates JIF with publication year [24].

With studies unmatched by topic area, and statistical significance as the outcome of interest, we observed a statistically significant association between JIF and study results: consistent with the results of previous studies [21, 22] however limited by confounding. With effect size rather than statistical significance as the measure of treatment effect, with matching for study characteristics, and with statistical adjustment for important quality-related confounders, the odds were uncertain. The estimated OR for greater than 50% mortality protection in high JIF journals was 2.5 (95% CI 0.6, 10), and the estimated odds of mortality in higher-JIF journals was 0.986 (95% CI 0.96, 1.02).

Also consistent with pre-existing studies, this study weakly supports the use of higher-JIF studies due to RCT design features that protect against bias. In the past, a study of among alcohol intervention trials found bivariate associations between study quality and JIF that were attenuated to inconsistent non-significance in their multi-predictor model [14]; in respirology, significance of the relationship between JIF and adequacy of allocation concealment remained with a small magnitude of association (OR 2.26) [15]. These design elements do not appear to predict future citations [25].

Conversely, a larger review of RCTs found a relatively large difference in the rate of adequate allocation concealment (66% vs 36%).The similar, small relative difference in blinding of providers (53% vs 41%) was also statistically significant [26]. Their comparison differed in that studies were unmatched by topic, analyses were unadjusted, the sample frame was a narrower journal set, and a higher threshold was set for “high” JIF.

As with this study, in bivariate analyses, higher-impact factor journals reported on RCTs with larger sample sizes, and studies more likely to be industry-funded [27]; this study also suggested a higher incidence of multicenter studies. In both this study and larger review, the trend was toward greater reporting of all-cause mortality as a primary outcome [26].

Whether or not a low-magnitude association truly exists between methodological quality and JIF, publication in high-impact journals appears non-discriminatory in selecting studies with design features that protect internal validity, with larger and historically more industry-funded studies being found in higher-impact journals [26]; however, the evidence remains very uncertain.

Together, the lack of association between JIF and study results, and the limited association between JIF and methodological quality, does not suggest that conservatively incorporating individual RCTs into practice would introduce significant bias in comparison to a systematic review of published RCTs. This assumes a similarly cautious approach, e.g., the use of non-interim publications [28]; a focus on mortality in this case, or to generalize, a similarly common and measurable outcome; and the use of study results independent of their statistical significance [29]. These published studies may present slightly greater effect estimates than those found in the grey literature [30]. Non-systematic data review may be more bias-prone [31], and a restricted approach to literature could sacrifice precision compared to systematic review. Rapidity of literature review and associated search restrictions exist on a spectrum: whereas physicians typically search for less than 2 min per question [32, 33], most published rapid reviews include grey literature and multiple databases, while including some literature search restrictions (e.g., on date or language [34]). Regardless, this study’s results would suggest that relevant, well-conducted primary research identified through rapid review through a search that is potentially JIF-biased can inform practice.

### Strengths and limitations

Several study strengths lend weight to this study and its conclusions. High-quality data formed the basis of the observation: RCTs with a consistent and reliable outcome, in a wide variety of topic areas selected randomly from a fairly unrestrictive sample frame. Matching of studies was performed rigorously and independently of us by content experts. Our statistical model also allowed for quantitative adjustment for study characteristics, aside from JIF, that may be associated with study results.

This study’s primary limitation is that its data represents a view of the literature from 15 years ago. Based on what we can infer, we do not anticipate that this limitation changed its primary conclusions about its null results. Publication bias has long been recognized; however, as high-JIF journals’ disseminated response is recent [1, 29], higher-JIF journals are probably publishing negative studies at least as often as they did previously. Thus, if time biased this study’s results, we expect a bias away from the null: (albeit speculatively) we would not expect that temporal effects nullified an otherwise observable association. Other limitations to this study’s generalizability arose from selecting the sample from systematic reviews of RCTs (which narrowed the sample frame), our restriction to RCTs published in English or in French, and removal of quasi-RCTs, which may have removed the better-reported quasi-randomized RCTs while keeping those that reported more poorly.

The primary threats to this study’s internal validity relate to its retrospective observational design. Though systematic reviews match similar RCTs, a degree of residual confounding is inevitable. The data suggested associations between factors related to internal validity and JIF [26] and between such factors and effect estimates. It is impossible to fully control for such confounders.

What further limits our statistical adjustments is that almost all of the investigated associations were imprecise, even where observed associations were of similar magnitude to associations that were statistically significant in earlier systematic reviews of similar numbers of trials [35, 36]. In choosing the mortality outcome, which rarely was a primary RCT outcome, this review selected an outcome that was rarely observed in many studies, which decreased this review’s power both to observe a difference in the primary outcome and to adjust precisely for confounders.

Also, as suggested by the protective association between mortality as primary outcome and estimated relative risk, the study of mortality as a non-primary outcome (91% of the sample) may not be representative of other outcomes: as it may be less prone to bias and less correlated to the reporting journal’s impact factor. Future research on JIF and bias should focus on trials’ primary outcomes both to improve its precision and to investigate the characteristics of the results that receive the focus of reporting and dissemination.

In terms of measurement, it would have been preferable to employ a pair of independent data extractors rather than one of the main investigators. Also, for the resolution of discrepancies, there was no advance calibration of methods experts with each other, and allocation concealment was assessed via an old scale. However, the data extractor’s training and experience combined with the input internationally recognized content experts supported the validity of the data extraction.

Although in broad terms we employed the current approach of domain-based evaluation of risk of bias, we did not separate the blinding of participants, personnel, and outcomes as per more recent Cochrane review guidelines [17]: neither did we systematically collect data around attrition bias, which qualitatively was extremely limited anyway in the studies’ reporting on their largely non-primary outcomes [17]. Adding the above-described details to the RCT descriptions would be unlikely to modify this study’s conclusions, however.

A conceptual limitation in interpreting this study is that the primary journal’s impact factor does not fully capture each RCT’s cumulative impact on clinical practice. JIF changes differently over time for different journals; however, its rate of change is low [37], and, over time, across the journals we included in our study, the relationship among journals’ journal impact factors was fairly stable (data available upon request). JIF does not account for secondary publications, conference presentations, guideline incorporation, lay media, and social media. Reporting on the content even within a primary paper is not homogeneous, as some results are emphasized more than others [38, 39]. Nonetheless, the impact factor of the original publishing journal appears to be a critical determinant of the frequency of subsequent citations [25, 27] and inexorably reigns as the most recognized principal measure of publication impact [40].

## Conclusion

In conclusion, study results seem not to vary with JIF, and the JIF may predict little in terms of methodological quality. The evidence is very uncertain. However, these observations would support the potential validity of readers’ unsystematic literature review: buttressing arguments for sometimes using rapid literature review to guide practice that is uninformed by a preexistent, up-to-date systematic review [41].

## Supplementary information

**Additional file 1.** PRISMA checklist.

**Additional file 2.**

## Data Availability

The datasets used and/or analyzed during the current study are available from the corresponding author on reasonable request.

## Abbreviations

CI: Confidence interval
JIF: Journal impact factor
OR: Odds ratio
RCT: Randomized controlled trial
RR: Relative risk

## Footnotes

1 First complete published report of the entire patient sample

2 Country of first author
